# TRPV1 Activation Exacerbates Hypoxia/Reoxygenation-Induced Apoptosis in H9C2 Cells via Calcium Overload and Mitochondrial Dysfunction

**DOI:** 10.3390/ijms151018362

**Published:** 2014-10-13

**Authors:** Zewei Sun, Jie Han, Wenting Zhao, Yuanyuan Zhang, Shuai Wang, Lifang Ye, Tingting Liu, Liangrong Zheng

**Affiliations:** Department of Cardiology, the First Affiliated Hospital, College of Medicine, Zhejiang University, No. 79 Qingchun Road, Hangzhou 310003, China; E-Mails: sns2006@zju.edu.cn (Z.S.); hanjie200641@163.com (J.H.); zhaowentingting@126.com (W.Z.); pizza_zyy@126.com (Y.Z.); wmmkao2@163.com (S.W.); a7451840@126.com (L.Y.); tingtingliu12345@163.com (T.L.)

**Keywords:** ischemia, apoptosis, calcium, mitochondrial, TRPV1 protein, rat

## Abstract

Transient potential receptor vanilloid 1 (TRPV1) channels, which are expressed on sensory neurons, elicit cardioprotective effects during ischemia reperfusion injury by stimulating the release of neuropeptides, namely calcitonin gene-related peptide (CGRP) and substance P (SP). Recent studies show that TRPV1 channels are also expressed on cardiomyocytes and can exacerbate air pollutant-induced apoptosis. However, whether these channels present on cardiomyocytes directly modulate cell death and survival pathways during hypoxia/reoxygenation (H/R) injury remains unclear. In the present study, we investigated the role of TRPV1 in H/R induced apoptosis of H9C2 cardiomyocytes. We demonstrated that TRPV1 was indeed expressed in H9C2 cells, and activated by H/R injury. Although neuropeptide release caused by TRPV1 activation on sensory neurons elicits a cardioprotective effect, we found that capsaicin (CAP; a TRPV1 agonist) treatment of H9C2 cells paradoxically enhanced the level of apoptosis by increasing intracellular calcium and mitochondrial superoxide levels, attenuating mitochondrial membrane potential, and inhibiting mitochondrial biogenesis (measured by the expression of ATP synthase β). In contrast, treatment of cells with capsazepine (CPZ; a TRPV1 antagonist) or *TRPV1* siRNA attenuated H/R induced-apoptosis. Furthermore, CAP and CPZ treatment revealed a similar effect on cell viability and mitochondrial superoxide production in primary cardiomyocytes. Finally, using both CGRP_8–37_ (a CGRP receptor antagonist) and RP67580 (a SP receptor antagonist) to exclude the confounding effects of neuropeptides, we confirmed aforementioned detrimental effects as *TRPV1*^−/−^ mouse hearts exhibited improved cardiac function during ischemia/reperfusion. In summary, direct activation of TRPV1 in myocytes exacerbates H/R-induced apoptosis, likely through calcium overload and associated mitochondrial dysfunction. Our study provides a novel understanding of the role of myocyte TRPV1 channels in ischemia/reperfusion injury that sharply contrasts with its known extracardiac neuronal effects.

## 1. Introduction

Transient receptor potential vanilloid type 1 (TRPV1), a ligand-gated cationic channel, is a molecular integrator of multiple chemical and physical stimuli, including high temperature, capsaicin (CAP) and tissue damage [1]. In the cardiovascular system, TRPV1 channels are localized in sensory nerves surrounding cardiovascular structures [1] as well as in cardiomyocytes [2,3]. We previously demonstrated that pharmacological activation of TRPV1 using CAP conferred a cardioprotective effect against ischemia/reperfusion injury [4] and that pharmacological inhibition or genetic deletion of TRPV1 enhanced ischemia/reperfusion injury [5]. This cardioprotective effect was attributed to the activation of TRPV1 channels on sensory neurons, leading to the release of key neuropeptides, including calcitonin gene-related peptide (CGRP) and substance P (SP) [6,7], both of which are known to be cardioprotective [8]. Moreover, we found that serum levels of CGRP and SP are decreased in patients with diabetes mellitus and coronary artery disease [9].

Although the aforementioned studies have highlighted the indirect cardioprotective effects of TRPV1 activation (mediated by stimulation of sensory neurons), recent studies show that TRPV1 activation on cardiomyocytes may exacerbate air pollutant-induced apoptosis [2,3]. Moreover, TRPV1 activation may also lead to apoptosis in normal cells such as retinal ganglion cells [10], synovial fibroblasts [11], breast cells [12] and dorsal root ganglion neurons [13] as well as tumors including osteosarcoma G292 cells [14], KB cancer cells [15], gastric cancer cells [16] and glioma cells [17]. Several mechanisms may contribute to TRPV1-related apoptosis, of which calcium influx plays a major role. A number of studies have shown that TRPV1 activation leads to an increase in intracellular calcium levels [11,14,18], which can result in calcium entry-dependent ROS production [11], mitochondrial depolarization and DNA fragmentation [19]. Increased calcium levels also caused activation of calpain, which participates in the mitochondrial apoptotic pathway through promoting Bid translocation to the mitochondria and nuclear translocation of apoptosis-inducing factor (AIF) [12]. Other mechanisms include endoplasmic reticulum stress and activation of the p38 MAPK pathway [17].

Despite major efforts to identify cardioprotective and pro-apoptotic pathways, the exact role of TRPV1 on cardiomyocytes during hypoxia/reoxygenation (H/R) remains unclear. In the present work, we set out to investigate the direct role of TRPV1 in H/R induced apoptosis in H9C2 cells. A major objective was to determine if TRPV1 activation *per se* is cardioprotective or deleterious. To that end, we used CAP and capsazepine (CPZ; a TRPV1 antagonist) to determine how TRPV1 activation modulates H/R-induced apoptosis. Intracellular calcium levels, mitochondrial function including mitochondrial superoxide production, mitochondrial membrane potential, and mitochondrial biogenesis (measured by the expression of ATP synthase β) were measured to determine the mechanism by which TRPV1 modulates apoptosis. Moreover, both genetic ablation of *TRPV1* using a siRNA strategy in H9C2 cells and treatment of primary cardiomyocytes with CAP or CPZ provided further evidence of the link between TRPV1 and H/R-induced apoptosis. Finally, using both CGRP_8–37_ (a CGRP receptor antagonist) and RP67580 (a SP receptor antagonist) to exclude the confounding effects of neuropeptides, we assessed the cardiac function in *TRPV1*^−/−^ and wide type mouse hearts to confirm the direct role of TRPV1 on cardiomyocytes during ischemia/reperfusion injury. Our results highlight the functional importance of direct TRPV1 activation on myocytes during H/R injury.

## 2. Results and Discussion

### 2.1. Results

#### 2.1.1. TRPV1 Is Expressed on H9C2 Cells and Activated during H/R

**Figure 1:**
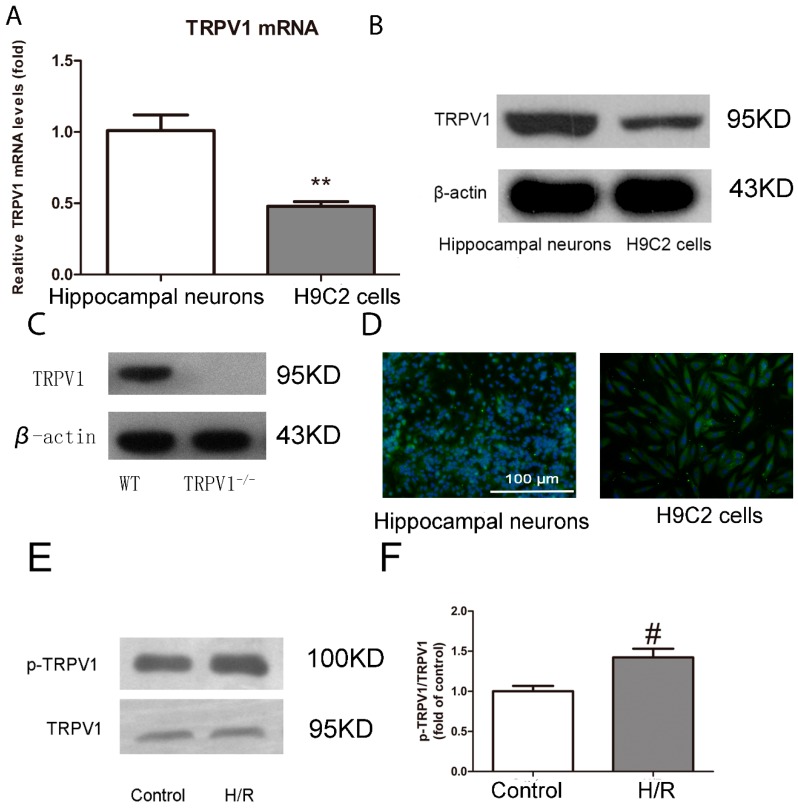
Transient receptor potential vanilloid type 1 (TRPV1) is expressed on H9C2 cells and activated during H/R. (**A**) Relative TRPV1 mRNA levels in H9C2 cells and hippocampal neurons are shown. Data are expressed as a percentage of TRPV1 mRNA levels in the hippocampal neurons group; (**B**) Western blots of TRPV1 in lysates obtained from H9C2 cells and hippocampal neurons; (**C**) Western blots of TRPV1 in lysates obtained from WT and *TRPV1*^−/−^ hearts; (**D**) Staining patterns of the TRPV1 in H9C2 cells and hippocampal neurons; (**E**) Protein expression of TRPV1 and phosphorylated TRPV1 in H9C2 cardiomyocytes in the presence or absence of H/R; (**F**) Quantitative summary of results from (**E**). *n* = 3. ** *p* < 0.01 *vs.* hippocampal neurons. # *p* < 0.05 *vs.* control.

Using a combination of RT-PCR, immunoblotting, and immunofluorescence microscopy, we investigated whether TRPV1 is expressed in H9C2 cells at the mRNA and protein levels. Hippocampal neurons were used as a positive control. As shown in Figure 1A, the mRNA expression levels of TRPV1 in H9C2 cells were approximately half of those in hippocampal neurons (*p* < 0.01). The level of TRPV1 protein expression in H9C2 cells was also weak compared with that in hippocampal neurons (Figure 1B). To demonstrate the specificity of the TRPV1 antibody, hearts from *TRPV1*^−/−^ mice were used as a negative control. As shown in Figure 1C, no TRPV1-positive band was detected in lysates from these hearts. Moreover, immunostaining and fluorescence microscopy revealed the presence of TRPV1-immunoreactivity on the surface of H9C2 cells and hippocampal neurons (Figure 1D).

To investigate whether TRPV1 was activated during H/R, we measured the protein expression levels of TRPV1 and phosphoTRPV1. Consistent with TRPV1 activation, challenge of H9C2 cells with H/R triggered the phosphorylation of TRPV1 (Figure 1E,F).

#### 2.1.2. Activation of TRPV1 Leads to the Loss of Cell Viability

To further confirm the involvement of TRPV1 in the response to H/R, cell viability assays were performed on H9C2 cells treated with various concentrations of CAP and CPZ. As expected, H/R reduced cell viability relative to control (normoxic) conditions. However, to our surprise, treatment with CAP induced a gradual decrease in cell viability, both with and without H/R challenge.

**Figure 2:**
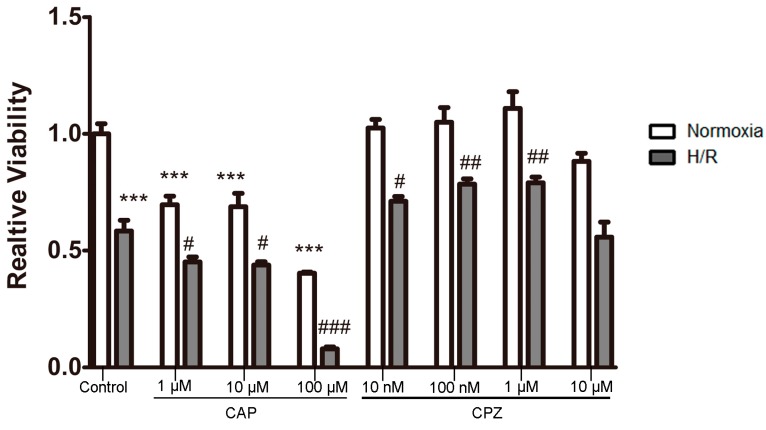
Activation of TRPV1 induces loss of cell viability. H9C2 cells were treated with various concentrations of CAP (1, 10, 100 μM) and CPZ (10 nM, 100 nM, 1 μM, 10 μM) in the presence or absence of H/R. Cell viability was measured using a MTT kit. *n* = 3. *** *p* < 0.001 *vs.* control + normoxia group. # *p* < 0.05, ## *p* < 0.01, ### *p* < 0.001 *vs.* control + H/R group.

In contrast, treatment of cells with CPZ at concentrations of 10 nM, 100 nM, or 1 μM significantly improved cell viability after H/R. However, at the highest concentration of CPZ that we tested, cell viability decreased significantly and was similar to levels achieved by the H/R challenge. Under normoxic conditions, cell viability was not altered at any drug concentration relative to control levels (Figure 2).

Taken together, our findings indicate that activation of TRPV1 during H/R worsens the effects of the H/R challenge.

#### 2.1.3. Activation of TRPV1 Induces Apoptosis and Elevates Intracellular Calcium Levels Following H/R

**Figure 3:**
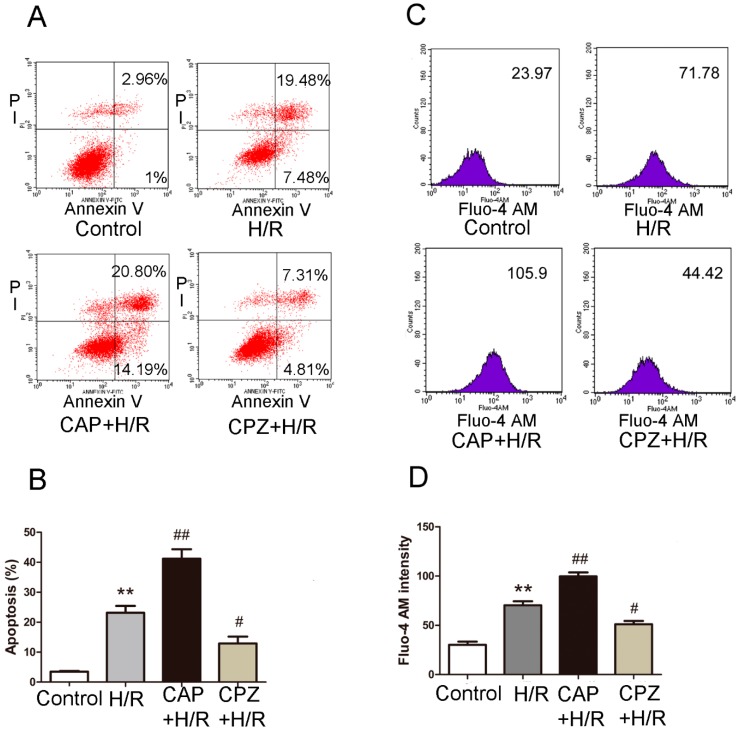
Activation of TRPV1 exacerbates apoptosis and increases intracellular calcium levels after H/R. H9C2 cells were treated with 1 μM CAP or 1 μM CPZ at the onset of H/R. Markers of apoptosis and intracellular calcium levels were measured by flow cytometry using annexin V/PI staining and Fluo-4 Acetoxymethyl (AM) staining, respectively. (**A**) The apoptotic H9C2 cells (annexin V+PI+ and annexin V+PI− cells) were analyzed. The numbers in each plot indicate the percentage of positive cells; (**B**) Analysis of results from three separate apoptosis experiments; (**C**) The mean Fluo-4 AM intensity of each group was analyzed; (**D**) Analysis of results from three separate calcium experiments. ** *p* < 0.01 *vs*. control. *# p* < 0.05, *## p* < 0.01 *vs*. H/R group.

We next tested whether TRPV1 activation triggers cell death. Shown in Figure 3A is the percentage of apoptotic H9C2 cells (annexin V+PI+ and annexin V+PI− cells) in each group. As expected, challenge of cells with H/R indeed triggered cell apoptosis. Treatment with 1 μM CAP exacerbated this effect, while 1 μM CPZ inhibited it (Figure 3B).

In other tissues, such as human airway epithelial cells and human bronchial epithelial cells, TRPV1 supports a strong Ca^2+^ conductance that leads to increased intracellular calcium [20,21], which in turn can promote apoptosis under stress conditions [22,23,24]. Therefore, we measured intracellular calcium influx in response to H/R challenge. As depicted in Figure 3C,D, exposure of H9C2 cells to H/R markedly increased intracellular calcium levels. Treatment with 1 μM CAP further aggravated this response, while 1 μM CPZ largely reversed it.

#### 2.1.4. Activation of TRPV1 Induces Mitochondrial Superoxide Production and Mitochondrial Membrane Depolarization

**Figure 4:**
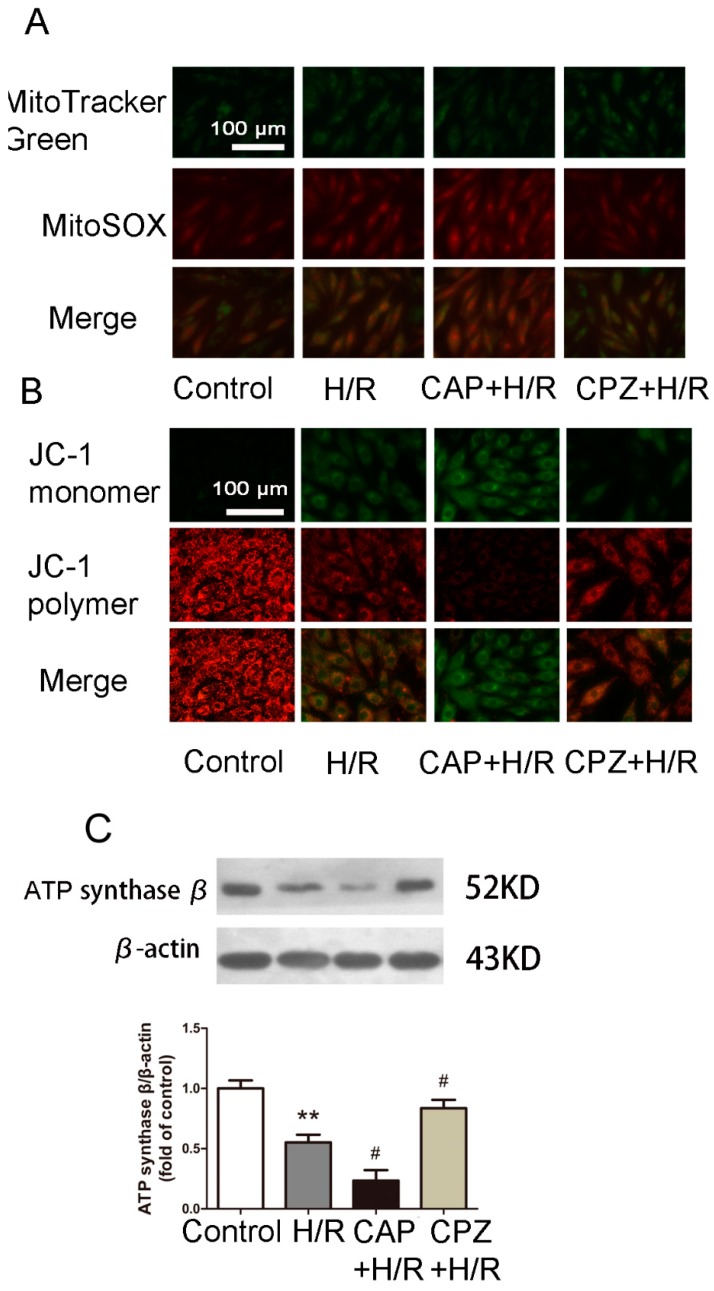
Activation of TRPV1 increases mitochondrial superoxide production, attenuates mitochondrial membrane potential, and inhibits mitochondrial biogenesis after H/R. H9C2 cells were treated with 1 μM CAP or 1 μM CPZ at the onset of H/R followed by MitoSOX Red staining, JC-1 staining, and Western blot analysis, (**A**) Mitotracker green and MitoSOX Red staining were used to track the mitochondrial and mitochondrial superoxide production, respectively; (**B**) Green and red fluorescence indicate depolarized and polarized mitochondrial membrane potentials, respectively; (**C**) The expression of ATP synthase β, a marker of mitochondrial biogenesis, was measured by Western blot analysis. *n* = 3. ** *p* < 0.01 *vs.* control. # *p* < 0.05 *vs.* H/R group.

Calcium influx into the cell is known to stimulate ROS production [25,26,27,28]. Whether TRPV1 activation further elevates or attenuates ROS levels remains unclear [29,30,31,32,33,34]. To address this, we measured mitochondrial superoxide production using MitoSOX Red. As expected, H/R induced an increase in mitochondrial superoxide production (Figure 4A). Treatment with 1 μM CAP caused further elevation in mitochondrial superoxide production, while 1 μM CPZ partially reversed this effect.

We further investigated the link between TRPV1 and mitochondrial membrane potential using JC-1 staining. As shown in Figure 4B, H/R reduced the mitochondrial membrane potential. Treatment with 1 μM CAP enhanced mitochondrial membrane depolarization, while 1 μM CPZ partly restored the mitochondrial membrane potential.

#### 2.1.5. Activation of TRPV1 Inhibited Mitochondrial Biogenesis

Mitochondrial biogenesis (MB) is often stimulated in an effort to synthesize new organelle constituents that aid in the replacement of dysfunctional mitochondria [35]. Therefore, we evaluated the efficiency of MB by detecting the expression of ATP synthase β, a known marker of MB [36,37]. As shown in Figure 4C, H/R induced a decrease of ATP synthase β expression. Treatment with 1 μM CAP further attenuated the expression of ATP synthase β, while 1 μM CPZ restored it back to basal levels.

#### 2.1.6. Knockdown of *TRPV1* by siRNA Inhibits Apoptosis and Improves Mitochondrial Function

**Figure 5:**
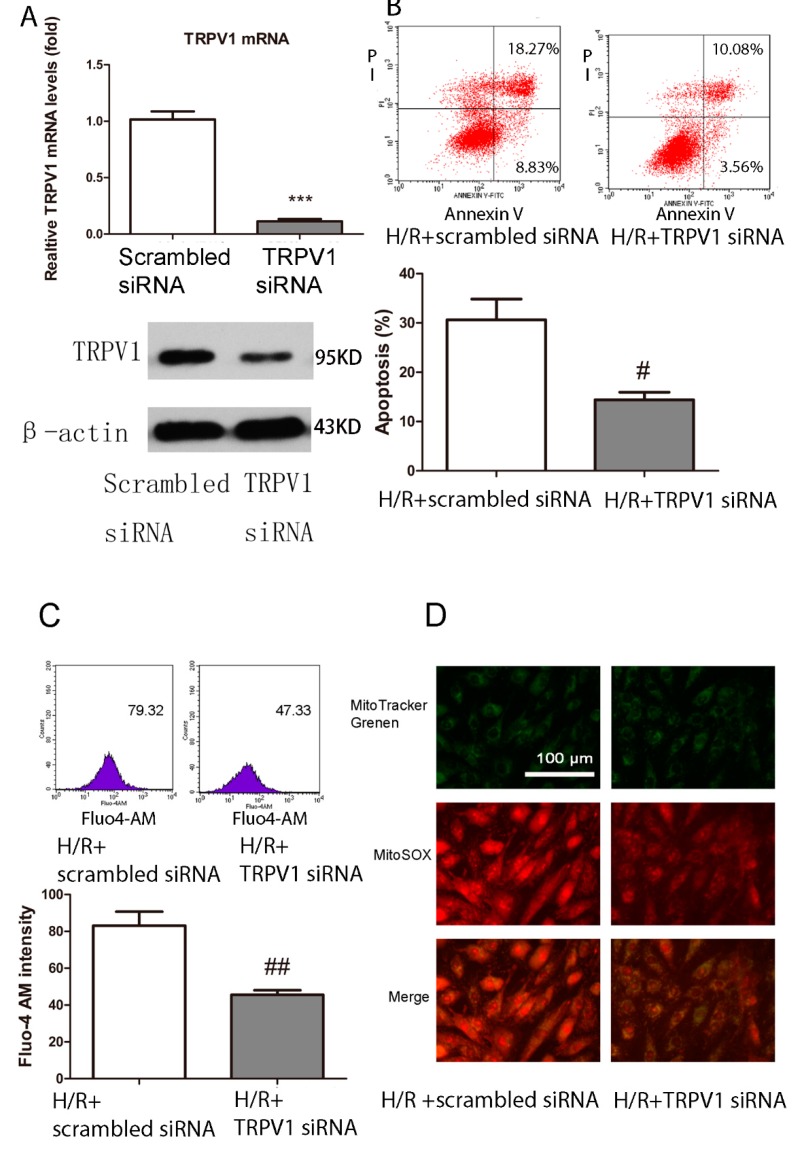
*TRPV1* siRNA ameliorates apoptosis and attenuates the intracellular calcium level and mitochondrial superoxide production after H/R. H9C2 cells were transfected with a scrambled siRNA or *TRPV1* siRNA before the onset of H/R. (**A**) Relative TRPV1 mRNA and protein levels were detected by RT-PCR and Western blot to show the transfection efficiency; (**B**) Apoptosis percentage of the two groups; (**C**) Intracellular calcium level of the two groups; (**D**) Mitochondrial superoxide production of the two groups. *n* = 3. *** *p* < 0.001 *vs.* scrambled siRNA group. # *p* < 0.05, ## *p* < 0.01 *vs.* H/R+ scrambled siRNA group.

To further confirm the involvement of TRPV1 in H/R-induced apoptosis, siRNA transfection was performed. As shown in Figure 5B, cells transfected with *TRPV1* siRNA revealed a cardioprotective effect, with a decrease of apoptosis compared with scrambled siRNA. Intracellular calcium influx and mitochondrial superoxide production were also inhibited compared with the scrambled siRNA group (Figure 5C,D).

#### 2.1.7. TRPV1 Activation Reduces Cell Viability and Increases Mitochondrial Superoxide Production in Primary Cardiomyocytes during H/R

**Figure 6:**
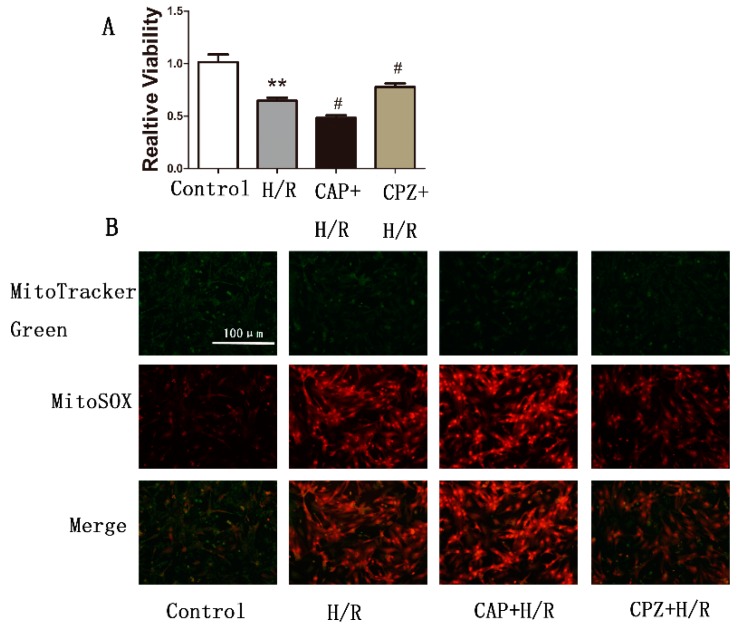
Activation of TRPV1 reduces cell viability and increases mitochondrial superoxide production. Cell viability and mitochondrial superoxide production were measured using an MTT kit and MitoSOX staining, respectively. (**A**) Cell viability and (**B**) Mitochondrial superoxide production of each group were analyzed. *n* = 3. ** *p* < 0.01 *vs.* control group. # *p* < 0.05 *vs.* H/R group.

Primary cardiomyocytes were isolated from neonatal wide type rats and CAP and CPZ were used to further confirm the role of TRPV1 in regulating cell viability and mitochondrial superoxide production. As shown in Figure 6, 1 μM CAP treatment reduced cell viability and increased mitochondrial superoxide production, while 1 μM CPZ partially reversed this effect.

#### 2.1.8. Improved Cardiac Function in *ex Vivo* *TRPV1*^−/−^ Hearts in the Presence of both CGRP_8–37_ and RP67580

**Figure 7:**
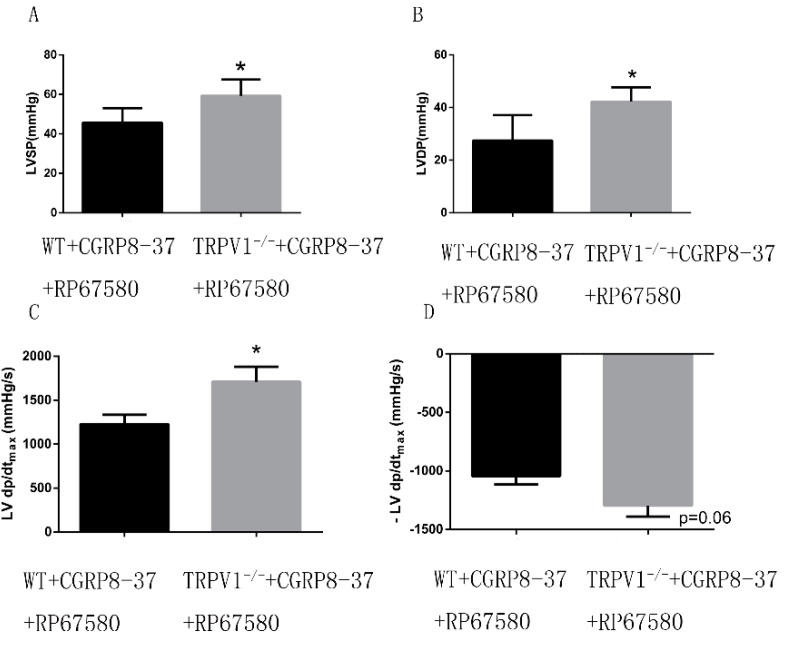
Improved cardiac function in *ex vivo* *TRPV1*^−/−^ hearts in the presence of CGRP_8–37_ and RP67580. *TRPV1*^−/−^ and WT hearts were retrogradely perfused in a Langendorff apparatus in the presence of both CGRP_8–37_ and RP67580 to block the effects of neuropeptides. Cardiac function, defined by the left ventricle systolic pressures (LVSP), left ventricle developed pressures (LVDP) and maximum speed of pressure development (±LV d*p*/d*t*_max_), were analysed. (**A**) LVSP of the two groups; (**B**) LVDP of the two groups; (**C**) +LV d*p*/d*t*_max_ of the two groups; (**D**) −LV d*p*/d*t*_max_ of the two groups. *n* = 8. * *p* < 0.05 *vs.* WT group.

Using both CGRP_8–37_ and RP6750 to block the effect of neuropeptides, we systematically assessed the cardiac function of *TRPV1*^−/−^ and widetype (WT) hearts to confirm the direct role of myocyte TRPV1 during ischemia/reperfusion. As shown in Figure 7, *TRPV1*^−/−^ hearts revealed an significant improvement of cardiac function, with an increase in left ventricle systolic pressures (LVSP), left ventricle developed pressures (LVDP) and +LV d*p*/d*t*_max_, while there was no significant difference in −LV d*p*/d*t*_max_ between these two groups (*p* = 0.06).

### 2.2. Discussion

The main findings of the present report are as follows: First, we found that TRPV1 is indeed expressed in H9C2 cells and activated during H/R injury, consistent with an important role for TRPV1 during H/R injury; Second, activation of TRPV1 channels by capsaicin promoted H/R-induced apoptosis of H9C2 cells, while treatment with capsazepine elicited a cardioprotective effect, indicating that myocyte TRPV1 exerts a deleterious effect during H/R injury; Third, CAP treatment triggered an increase in intracellular calcium levels leading to increased mitochondrial superoxide production and depolarization of the mitochondrial membrane potential. These effects were inhibited by CPZ, indicating that calcium overload and mitochondrial dysfunction were mechanistically involved in TRPV1-induced apoptosis; Fourth, activation of TRPV1 inhibited mitochondrial biogenesis, while CPZ treatment restored it to basal levels, highlighting the importance of TRPV1 in the regulation of mitochondrial function; Finally, the deleterious role of TRPV1 was further identified by the transfection of *TRPV1* siRNA, which promoted apoptosis, likely by increasing intracellular calcium and mitochondrial superoxide levels.

The present findings contrast with our previous work, in which we demonstrated that TRPV1 activation with CAP treatment elicited a cardioprotective effect against ischemia/reperfusion injury [4], while deletion of TRPV1 (either using an antagonist or *TRPV1* knockout mice) enhanced damage after myocardial infarction [5]. We hypothesize that this difference may be explained by neuropeptide release *in vivo*. Indeed, activation of TRPV1 can stimulate the sensory neurons to release key neuropeptides, including CGRP and SP [6,7], which are known to protect against ischemic injury [8,38,39]. However, in this study, with the absence of the confounding effects of neuropeptides, activation of TRPV1 revealed its direct effect on H9C2 cells during H/R, which induced apoptosis. In fact, in many cancer cells, such as nasopharyngeal carcinoma NPC-TW 039 cells, A172 human glioblastoma cells and urothelial cancer cells [17,32,40], TRPV1 activation also produces a pro-apoptotic effect.

Like other members of the TRP family, TRPV1 activation can lead to a potent influx of extracellular calcium [20,21,41]. This, in turn, induces a variety of intracellular events, including apoptotic cell death in epithelial cells of the lung [20,21] and in microglia of the brain [41]. Hence, we investigated the link between intracellular calcium levels and TRPV1 induced apoptosis. We found that CAP, which increases calcium levels, likely promotes apoptosis through intracellular calcium overload.

Consistent with previous reports [42], mitochondrial dysfunction was another key factor leading to H/R-induced apoptosis. Although increased intracellular calcium levels promote ROS generation [25,26,27,28], the induction of ROS by TRPV1 activation was confusing. While activation of TRPV1 is known to trigger ROS production [29,30,31], in recent studies TRPV1 activation was shown to induce apoptosis by inhibiting ROS production [32,33,34], suggesting that overproduction as well as suppression of ROS generation may play a role in CAP-induced apoptosis. In our study, TRPV1 activation led to ROS production, indicating that TRPV1 aggravated the H/R-induced apoptosis via increasing ROS production.

We measured changes in the mitochondrial membrane potential as a key determinant of mitochondrial function. Unlike ROS production, the link between TRPV1 and the mitochondrial membrane potential was relatively clear as CAP treatment has been previously shown to induce mitochondrial membrane potential collapse [11]. Our data was in agreement with these previous findings. Based on our data on ROS production and mitochondrial membrane potential, we propose that the activation of TRPV1 aggravates apoptosis via inducing mitochondrial dysfunction.

These data are consistent with the notion that activation of TRPV1 exacerbates mitochondrial damage. Once damaged, mitochondrial biogenesis (MB) may be stimulated in order to synthesize new organelle constituents followed by the integration of these components (*i.e.*, proteins and lipids) into preexisting mitochondria [35]. ATP synthase β, one of the nDNA-encoded OXPHOS proteins is synthesized and imported into mitochondria [36,37] and is regarded as a marker of MB. We further investigated the association of TRPV1 and MB by measuring the expression of ATP synthase β. H/R significantly decreased the expression of ATP synthase β, which was consistent with the results in an ischemia–reperfusion acute kidney injury model [43]. Furthermore, activation of TRPV1 inhibited the MB, while CPZ enhanced the MB, indicating that activation of TRPV1 not only induced mitochondrial dysfunction, but also inhibited mitochondrial recovery thus aggravating H/R-induced apoptosis.

We further confirmed the direct role of TRPV1 on cardiomyocytes in *TRPV1* siRNA transfected H9C2 cells, primary cardiomyocytes and *TRPV1*^−/−^ mice. Indeed, genetic silencing of *TRPV1* attenuated apoptosis, inhibited intracellular calcium levels and mitochondrial superoxide production. Furthermore, CAP treatment reduced cell viability and increased mitochondrial superoxide production in primary cardiomyocytes while CPZ treatment partially reversed this effect. In addition, improvement of cardiac function was also seen in *TRPV1*^−/−^ mouse hearts when the effects of neuropeptides were blocked. These results confirmed that TRPV1 plays a deleterious role in H/R induced apoptosis.

Our study has several limitations that require mention. First, activation of TRPV1 might lead to the release of other neuropeptides, which remains unknown, thus possible effects of neuropeptides cannot be completely ruled out by treatment with CGRP_8–37_ and RP67580. Second, the apoptosis pathway was not thoroughly examined and in particular, the link between TRPV1 and activation of caspases should be further investigated.

## 3. Experimental Section

### 3.1. Reagents

Capsaicin (CAP) and capsazepine (CPZ) were purchased from Sigma–Aldrich (St. Louis, MO, USA). MTT Cell Proliferation and Cytotoxicity Assay Kit were purchased from Beyotime (Shanghai, China). Annexin V-FITC Apoptosis Detection Kit was purchased from BD (Bedford, MA, USA). Antibody against TRPV1 was purchased from Millipore (Billerica, MA, USA). Antibody against phosphoTRPV1 was purchased from Wako Pure Chemical Industries (Osaka, Japan). Antibody against ATP synthase β was purchased from Abcam (Cambridge, UK). Antibody against β-actin was purchased from CST (Danvers, MA, USA). Dulbecco’s Modified Eagle’s Medium, fetal bovine serum, Fluo-4 AM, MitoTracker Green FM, MitoSOX Red Mitochondrial Superoxide Indicator, JC-1, and Lipofectamine RNAi MAX Transfection Reagent were obtained from Life Technologies (Grand Island, NY, USA). CGRP_8–37_ and RP67580 were obtained from Tocris Bioscience (Bristol, UK).

### 3.2. Cell Culture

Rat heart tissue-derived H9C2 cardiac myoblast cell line was purchased from American Type Culture Collection (ATCC, Manassas, VA, USA). Cells were cultured in Dulbecco’s modified Eagle’s medium (DMEM) supplemented with 10% fetal bovine serum (FBS). Treatment with CAP (at concentrations of 1, 10, and 100 μM) and CPZ (at concentrations of 10 nM, 100 nM, 1 μM and 10 μM) were accomplished by adding these compounds at the onset of H/R.

Primary cultures of hippocampal neurons, which express the TRPV1 channel [44,45], were used as a positive control. These neuronal cells were obtained from Dr. Luo and cultured as previously described [46].

Primary cardiomyocytes were isolated from the whole heart of 1–2 day old rats as described before [47]. Briefly, hearts were minced, digested with trypsin overnight at 4 °C. The tissue was then dissociated by stepwise collagenase treatment for a few minutes at 37 °C. Cells were pre-plated twice for 60 min to eliminate fibroblasts. The non adherent myocytes were then plated in plating medium consisting of 199 medium supplemented with HEPES, MEM non-essential amino acids, glucose, glutamine, 10% FBS, vitamin B12, penicillin and streptomycin, on fibronectin coated plates. Following overnight culture, cells were washed and fresh medium with 2% FCS was added.

### 3.3. Hypoxia/Reoxygenation

The control cultures were maintained in normoxia (21% O_2_, 5% CO_2_) throughout. An H/R model was set up using a hypoxia controlled chamber as previously described [42]. Briefly, H9C2 cell cultures were subjected to hypoxia (1% O_2_, 5% CO_2_, 94% N_2_; 5 h) followed by 1 h of reoxygenation (21% O_2_, 5% CO_2_).

### 3.4. Cell Viability

Cell viability was measured using a MTT Cell Proliferation and Cytotoxicity Assay Kit (Beyotime, Shanghai, China). Briefly, H9C2 cells were either challenged with H/R or incubated under normoxic conditions with the presence of various concentrations of CAP or CPZ. Then cells in each well were incubated with MTT solution (0.5 mg/mL) for 4 h. Finally, 100 μL of DMSO was added, and the absorbance was read at 590 nm using an automatic microplate reader (Bio-Tek, Winooski, VA, USA).

### 3.5. Cell Apoptosis

Apoptosis was detected using a standard assay. In brief, cells were resuspended and incubated with 5 μL of Ann-V and 5 μL of PI for 15 min in the dark. Samples were analyzed by flow cytometry, and the results are presented as the percentage of cells that were viable (Annexin V−PI−), early apoptotic (Annexin V+PI−), late apoptotic (Annexin V+PI+) or nonviable Ann-V−PI+). Finally, the percentage of early and late apoptotic cells in each group was compared.

### 3.6. RT-PCR

Total RNA was isolated from H9C2 cells with TRIzol (Life Technologies) according to the manufacturer’s protocol and cDNA was synthesized using RvertAid First Strand cDNA Synthesis Kit (Fermentas, Vilnius, Lithuania). RT-PCR reactions were performed using 3 μL diluted cDNA product as described previously [48]. Primers used for RT-PCR were: *TRPV1* 5'-GAAGCAGTTTGTCAATGCCAGCTA-3' (forward), 5'-AGGGTCACCAGCGTCATGTTC-3' (reverse); β-actin 5'-CGTTGACATCCGTAAAGACC-3' (forward), 5'-TAGAGCCACCAATCCACACA-3' (reverse).

### 3.7. Western Blot Analysis

H9C2 cells were lysed in RIPA buffer containing a cocktail of protease and phosphatase inhibitors. Total protein content was measured using the BCA assay. Twenty micrograms of total protein were loaded into SDS-PAGE gels and immunoblots were performed as previously described [49]. The concentrations of all the antibodies, including TRPV1, phosphorylated TRPV1, ATP synthase β, and β-actin, were set at 1:1000.

### 3.8. Immunofluorescence Analysis

To determine the expression of TRPV1, H9C2 cells and hippocampal neurons (as a positive control) were fixed and permeabilized using CytoFix/CytoPerm Plus (BD). Cells were then incubated with the antibody against TRPV1 (1:200 dilution) overnight at 4 °C. Goat anti-mouse IgG H&L (FITC) secondary antibody was used at a 1/200 dilution for 1 h. Nuclei were counter-stained with DAPI (blue). All samples were analyzed using a fluorescence microscope (Nikon, Tokyo, Japan).

### 3.9. Intracellular Calcium Level Measurement

Intracellular Ca^2+^ flux was measured using Fluo-4 AM staining according to the manufacturer’s protocol. Briefly, cells were re-suspended in a calcium and magnesium-free PBS/glucose medium supplemented with 5 μmol/L Fluo 4-AM and incubated in the dark for 30 min at 37 °C. After washing, cells were incubated for a further 30 min to allow complete de-esterification of intracellular AM esters. Fluo-4 AM fluorescence was measured using a flow cytometer.

### 3.10. Determination of Mitochondrial Superoxide Production

Mitochondrial superoxide production was measured by fluorescence microscopy using the chemical probe, MitoSOX Red. In brief, cells were loaded with 2.5 μM MitoSOX Red for 30 min at 37 °C. Cells were then treated for 15 min with 200 nM MitoTracker green to stain mitochondria. After washing with PBS, cells were examined under a fluorescence microscope.

### 3.11. Assessment of Mitochondrial Membrane Potential

To measure the mitochondrial membrane potential (ΔΨm), JC-1, a sensitive fluorescent probe for ΔΨm, was used. At the end of the specific experimental procedure, H9C2 cells were rinsed with PBS twice and stained with 5 μM JC-1 for 30 min at 37 °C. Cells were rinsed with PBS twice and immediately analyzed by fluorescence microscopy. Mitochondrial depolarization was indicated by an increase in the red/green fluorescence intensity ratio.

### 3.12. siRNA Transfection

H9C2 cells with >50% subconfluency were transfected with *TRPV1* siRNA (Biomics Biotech, Nantong, China) using Lipofectamine RNAi MAX Transfection Reagent. All experiments were performed 48 h after transfection.

### 3.13. Animal Preparation

Male *TRPV1* gene knockout (*TRPV1*^−/−^) mice were obtained from Jackson Laboratory (Bar Harbor, ME, USA) as described before [5]. Wide-type (WT) C57BL/6 mice were obtained from Shanghai Laboratory Animal Center of the Chinese Academy of Sciences (Shanghai, China). All mice were 8 weeks old with an average weight of 25–30 g and housed in the animal facility, which was maintained at 20–25 °C, 55% relative humidity, and with an automatic 12-h light/dark cycle. All mice were allowed to acclimate for 1 week before the experiments started and received a standard laboratory diet and tap water *ad libitum*. All the procedures were approved by the Animal Care and Use Committee of Zhejiang University (Register ID No.: ZJU20140112009; Date: 15 January 2014), and conformed to the Guide for the Care and Use of Laboratory Animals published by the US National Institutes of Health (NIH Publication No. 85-23, revised 1996).

### 3.14. Langendorff Heart Preparation and Measurements of Cardiac Function

The hearts were cannulated on the Langendorff apparatus system and perfused with the Krebs–Henseleit buffer (NaCl 118 mmol/L, KCl 4.7 mmo/L, MgSO_4_ 1.2 mmol/L, KH_2_PO_4_ 1.2 mmol/L, CaCl_2_ 2.5 mmol/L, NaHCO_3_ 2.5 mmol/L, Na-EDTA 0.5 mmol/L, glucose 11 mmol/L, pH 7.4, saturated with 95% O_2_ and 5% CO_2_) retrogradely from the aortic root. A water-filled balloon was set up, which was connected to the Medlab 6.0 system (Nanjing Meiyi Science and Technology Co., Ltd., Nanjing, China) through the catheter and pressure transducer. The balloon was inserted into the left ventricle and the left ventricular end-diastolic pressure (LVEDP) was adjusted to between 5 and 8 mmHg through controlling the balloon volume so that the Medlab system was able to measure the heart function precisely.

Hearts were paced at 350 bpm except during ischemia, and pacing was reinitiated 2 min after reperfusion. After a 25-min equilibration period, hearts were subjected to 40 min of no-flow normothermic global ischemia, followed by 30 min of reperfusion. Both CGRP_8–37_ (1 µmol/L) and RP67580 (1 µmol/L) were added to the perfusate 5 min before ischemia. The left ventricle systolic pressures (LVSP), left ventricle developed pressures (LVDP), and maximum speed of pressure development (±LV d*p*/d*t*_max_) were measured during the process.

### 3.15. Statistical Analysis

Data are expressed as mean ± SEM. In cellular experiments, all preparations, experiments, and measurements were repeated at least three times. For animal experiments, at least 8 measurements were averaged. Statistical analyses were performed with an unpaired Student’s *t* test after the demonstration of homogeneity of variance with an F test or a one-way ANOVA for more than two groups. *p*-values less than 0.05 were considered statistically significant.

## 4. Conclusions

In conclusion, TRPV1 channels modulate the severity of ischemia-reperfusion injury through direct and indirect effects that elicit divergent responses. While TRPV1-mediated neuropeptide release from sensory neurons is indeed cardioprotective, our current findings indicate that its activation on myocytes promotes apoptosis. The underlying mechanism likely involves intracellular calcium overload and associated mitochondrial dysfunction, including inhibition of mitochondrial biogenesis. Pharmacological inhibition, genetic silencing and gene deletion all revealed a potent cardioprotective role for TRPV1. These findings establish a previously unrecognized direct role of TRPV1 on cardiomyocytes in the modulation of ischemia-reperfusion injury.
